# Enhanced Ionic Conductivity and Electrochemical Properties of Li_2_B_12_H_12_/ZrO_2_ Nanocomposites for All-Solid-State Lithium Metal Batteries

**DOI:** 10.1021/acsami.5c01939

**Published:** 2025-06-02

**Authors:** Jonas D. Hehn, Hendrik P. Rodenburg, Masoud Lazemi, Juliette C. Verschoor, Marta Perxés Perich, Martin Sundermann, Hlynur Gretarsson, Jessi E. S. van der Hoeven, Frank M. F. de Groot, Petra E. de Jongh, Peter Ngene

**Affiliations:** † Materials Chemistry and Catalysis, Debye Institute for Nanomaterials Science, 8125 Utrecht University, Utrecht 3584 CG, The Netherlands; ‡ 28332 Deutsches Elektronen-Synchrotron DESY, Hamburg 22607, Germany; § Max Planck Institute for Chemical Physics of Solids, Dresden 01187, Germany

**Keywords:** solid-state electrolyte, all-solid-state battery, nanocomposites, interface engineering, nanostructuring, metal hydrides

## Abstract

Solid-state electrolytes play a key role in the development of safe and high-capacity all-solid-state batteries. Complex hydrides such as Li_2_B_12_H_12_ are attractive as solid electrolytes due to their low weight and good electrochemical stability, but suffer from low conductivities at room temperature. Herein, we report a three-order-magnitude increase in the ionic conductivity of Li_2_B_12_H_12_ upon nanocomposite formation with ZrO_2_ via mechanochemical treatment, reaching 2.9 × 10^–4^ S cm^–1^ at 30 °C. Results from infrared spectroscopy, X-ray Raman scattering and electron microscopy coupled with electron energy loss spectroscopy suggest that the increased ionic conductivity is due to strong interfacial interaction/reaction between Li_2_B_12_H_12_ and ZrO_2_. This leads to a highly defective interphase region where the Li, B, Zr, and O chemical environments are distinctively different from the bulk Li_2_B_12_H_12_ and ZrO_2_. The improved ionic conductivity of the nanocomposite compared to the pristine material enabled the realization of all-solid-state batteries with a Li metal anode and both TiS_2_ and LiFePO_4_ cathodes. We demonstrate the suitability of the nanocomposite at various charging rates up to C/2 (0.34 mA cm^–2^) for over 170 cycles at 40–60 °C (Li|Li_2_B_12_H_12_/ZrO_2_|TiS_2_).

## Graphical abstract

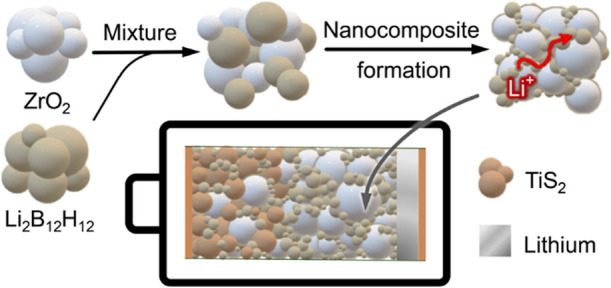

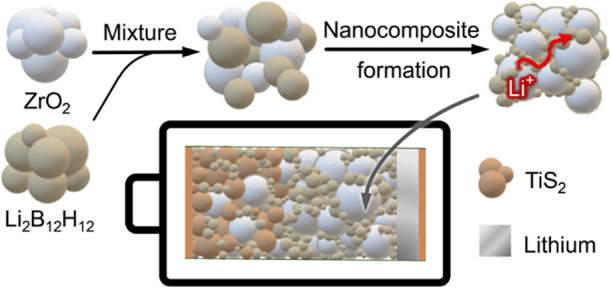

## 1 Introduction

The development of electrochemical energy storage technologies is one of the greatest innovations in recent decades, as evidenced by their use in a wide range of electronic devices and the award of the 2019 Nobel Prize to researchers in the field of Li-ion batteries.^1^ However, most commercially available batteries contain liquid electrolytes, limiting the energy density and posing safety concerns like leakage and flammability.^2–4^ The development of all-solid-state batteries (ASSBs) could overcome these barriers or drawbacks by using solid-state electrolytes (SSEs). A wide chemical stability window and intrinsic rigidity of solid electrolytes could potentially prevent the growth of dendrites, thereby allowing new battery chemistries with lithium metal anodes.^5–7^

Various types of SSEs have been introduced in the past years. In addition to the well-explored oxidic and sulfide-based compounds, complex metal hydrides (e.g., LiBH_4_, Li_2_B_12_H_12_) have recently been shown to be a promising class of SSEs.^8–10^ Properties like low density, high deformability and compatibility with lithium metal result in effective interface formation with electrodes and enable the fabrication of high-energy-density batteries.^11^ Li_2_B_12_H_12_ possesses further attributes like being nonflammable and exhibiting impressive Li-ion conductivity following a polymorphic transition at ∼355 °C.^12^ However, its utilization in ASSBs will require high ionic conductivity (∼10^–3^ S cm^–1^) at moderate (i.e., 55 °C)^10^ to room temperature. Ye et al.^13^ solution cast PVDF-HFP-PEO-LiTFSI-Li_2_B_12_H_12_ and successfully operated a LiFePO_4_ cathode and lithium metal anode cell combined with the obtained composite solid electrolyte, while also investigating the role of residual solvent. In addition to techniques like dissolution or embedding in a polymer matrix,^13,14^ approaches such asnanostructuring^12,15^ and nanoconfinement/interface formation with oxide scaffolds^16–19^ have also been explored and found effective in improving ionic conductivity.

Nanocomposite formation or interface engineering is an interesting approach because, in addition to improved ionic conductivity, the presence of the inorganic additive can improve the electrochemical properties of the SSE, such as the interface stability and the electrochemical stability window. This is due to improved mechanical properties and favorable interaction between the additive and the electrolyte materials. For instance, Zhou et al.^16^ recently demonstrated that the mixing of Al_2_O_3_ and Li_2_B_12_H_12_ leads to a nanocomposite electrolyte with improved electrochemical stability and enables a 3.8 V ASSB with In/Li alloy as anode and NMC (lithium nickel manganese cobalt oxide) cathode material. However, the origin of the increased conductivity was not discussed. Inspired by this, and the fact that the enhancement in ionic conductivity and electrochemical properties of nanocomposite solid electrolytes strongly depends on the properties of the metal oxide additive, we investigate the ionic conductivity and electrochemical properties of Li_2_B_12_H_12_/ZrO_2_ nanocomposites. ZrO_2_ was chosen due to its intriguing properties, such as accessible meso- and microporosity and the presence of strong acid sites, which have been linked to the interface interactions that lead to very high ionic conductivity in LiBH_4_.^3,17,20^

We show that ball milling a mixture of ZrO_2_ and Li_2_B_12_H_12_ yields nanocomposites with conductivities up to 3 orders of magnitude higher than pristine Li_2_B_12_H_12_, exhibiting 2.9 × 10^–4^ S cm^–1^ at 30 °C. Moreover, we demonstrate their suitability for ASSB applications. To unravel the origin of the conductivity enhancement, we probed electronic and structural changes, revealing that it can be attributed to the formation of a tertiary compound or an interphase at the interface between the two compounds.

## 2 Experimental Section

### 2.1 Synthesis

Li_2_B_12_H_12_·4H_2_O (>98%, KatChem) was dried at 230 °C for 18 h under reduced pressure (1 mbar) to remove lattice water. Zirconia ZrO_2_ (RC 100, Gimex) with a Brunauer–Emmett–Teller (BET) surface area of 95 m^2^ g^–1^ and a total pore volume of 0.246 cm^3^ g^–1^ was dried at 280 °C under reduced pressure (1 mbar) to remove physisorbed water and was subsequently mixed with the dehydrated Li_2_B_12_H_12_ in different ratios (Table 1). The Li_2_B_12_H_12_:ZrO_2_ ratios are expressed in terms of vol %, wt % and pore filling fraction%, which is the ratio of the volume of Li_2_B_12_H_12_ added in the mixture to the total pore volume of the ZrO_2_. Above 100% pore filling, the amount of complex metal hydride used is higher than the interparticle pore volume of the oxide. Hence, a continuous Li-ion pathway is ensured. Samples with a pore filling fraction of less than 100% are synthesized to exclusively probe the interface layer, which becomes more dominant as the contribution of the bulk-like Li_2_B_12_H_12_ is excluded or minimized. The samples were handled in an argon-filled glovebox (MBraun, Lab Star Glove Box, <1 ppm of O_2_ and <1 ppm of H_2_O), to prevent reaction with water. All samples were prepared by ball milling (BM) 20–90 h in 20 mL tungsten carbide grinding bowls at 400 rpm in a Fritsch Pulverisette 7 for milling periods of 22 min separated by 18 min breaks to prevent thermally induced decomposition of the Li_2_B_12_H_12_. Balls with a diameter of 5 mm made from tungsten carbide were used for the mechanochemical treatment. The ball-to-sample weight ratio was kept constant at 35:1 and an absolute amount of sample between 300–750 mg was used per batch. After ball milling, the samples were taken into the glovebox for further handling and storage.

**Table 1:** Composition and Fraction of Pore Filling of the Investigated Samples

Sample	oxide fraction (vol %)	oxide fraction (wt %)	pore filling fraction (%)
25 vol % ZrO_2_	25	62.5	207
50 vol % ZrO_2_	50	83.3	69
75 vol % ZrO_2_	75	93.8	23

### 2.2 Characterization

#### 2.2.1 Structural Characterization

The crystallographic structure of the composites was measured with powder X-ray diffraction (XRD) on a Bruker D2 Phaser equipped with a Cobalt source (Co K_α1,2_ radiation, λ = 1.79026 Å) at 30 kV and 10 mA. Diffractograms were measured at room temperature from 2θ = 10° to 60° in an argon-filled sealed specimen holder. Nitrogen physisorption measurements were carried out to analyze the porosity of the oxide filler material. The sample was first dried at 285 °C under dynamic vacuum, and subsequently the measurement was carried out at 77 K in a TriStar II Plus gas-volumetric instrument (Micromeritics). The BET surface area was obtained by fitting the experimental data with a BET isotherm (0.05 < *p*/*p*_0_ < 0.25).^21^ The interparticle pore volume of ZrO_2_ was determined from the volume of absorbed N_2_ at *p*/*p*_0_ = 0.995. In order to determine the effects of the material of the ball mill jar and balls (88% tungsten carbide, 12% cobalt) on the nanocomposite, elemental analysis with inductively coupled plasma (ICP) measurement was conducted on the nanocomposite. This was done at the Mikroanalytisches Laboratorium Kolbe in Oberhausen, Germany. Diffuse reflectance infrared Fourier transform spectroscopy (DRIFTS) measurements were recorded on a Nicolet iS50 FT-IR spectrometer equipped with a Praying Mantis Accessory (Harrick). The samples were mixed with dry FT-IR grade KBr (≥99% Sigma-Aldrich) and placed in an argon-filled sample holder with KBr windows. Data was collected using a liquid nitrogen-cooled MCT detector from 4000 to 1000 cm^–1^ with a 4 cm^–1^ resolution, averaged over 64 scans. Kubelka–Munk transformation was used to analyze the data.^22^ Samples for differential scanning calorimetry (DSC) were prepared in aluminum crucibles that allow gas exchange. For the measurement, a Mettler Toledo HP DSC 1 with a PC10 pressure controller element was used. DSC data was collected between 30 and 370 °C with a heating/cooling rate of 10 K min^–1^, under an argon pressure of 2 bar and a continuous argon flow of 10 mL min^–1^. Scanning electron microscopy (SEM) images were acquired using a Zeiss Evo 15 microscope.

Scanning transmission electron microscopy combined with electron energy loss spectroscopy (STEM-EELS) was performed in a probe-corrected Spectra 300 microscope (Thermo Fischer Scientific) operated at 300 kV in STEM mode. Dual-EELS data was acquired with a CCD camera using DigiScan in the Gatan Microscopy Suite, with a dispersion of 0.15 eV/pixel, a convergence angle of 16.0 mrad, a collection semiangle of 12.88 mrad and an entrance aperture of 5 mm. Energy dispersive X-ray (EDX) spectra were recorded with a screen current of 94 pA, 461 × 459 pixels with a size of 0.1092 nm per pixel, a dwell time of 40 μs and 104 frames. Boron (B) and lithium (Li) K-edge X-ray Raman scattering (XRS) spectra were measured at the P01 beamline of the Deutsches Elektronen-Synchrotron (DESY) Petra III, in Hamburg, Germany. Typically 50–100 mg sample was compacted into a pellet with 10 mm diameter and placed in an airtight XRS cell described elsewhere.^20^ The XRS measurement was operated in reflectance mode with an incidence beam angle of 15.8°. A scattered photon energy of 9.69 keV was selected using the Si(660) reflection of 12 spherically bend analyzers centered around 2θ = 55°, resulting in an average *q*-vector of 4.5 Å^–1^, and detected using a Medipix-3 based pixel detector. Spectra were then taken by scanning the incident energy using a Si(311) double crystal monochromator, with an overall energy resolution of 0.7 eV. At this scattering angle, XRS is essentially in the dipole limit and the XRS spectrum will be equivalent to the X-ray absorption spectrum (XAS). In the case of the measured Li and B K-edges the XRS = XAS spectrum probes the empty density of states. To avoid beam-induced sample damage during the measurement, the beam-exposed sample area was changed every 3 min. The regions of interest (ROI) containing the scattered signal from the sample, were determined/analyzed manually. The background was removed with a Python-based XRStools software by utilizing the parametrized Hartee–Fock Compton profiles and PearsonVII functions.^23^ Independently, we validated the background subtraction by fitting the Compton profile with a log-normal function, which led to similar results. In addition, the data was smoothened by adjacent averaging over five points and normalized.

#### 2.2.2 Electrochemical Characterization

All electrochemical measurements were carried out in custom-made cells (Figure S1) with a Parstat MC multichannel potentiostat equipped with PMC-1000 (Ametek). The impedance data was used to calculate the conductive properties of the SSE samples. Electrochemical impedance spectroscopy (EIS) was measured at 20 mV AC from 1 MHz to 1 Hz. Typically, 50 mg powder was compacted between two 10 mm stainless-steel rods and pressurized with 187 MPa to form a pellet with a thickness of about 0.3–0.5 mm, before the cell was tightened with a torque of 2.5 N m. The cells were placed in a UF55plus oven (Memmert) to record EIS data from 30 to 100 °C in 10 °C increments. Impedance data was fitted using the Python-based software DECiM^24^ to extract the total electrolyte resistance *R*_electrolyte_ = *R*_bulk_ + *R*_gb_, where *R*_bulk_ is the resistivity of the bulk material, and *R*_gb_ describes a contribution that could be attributed to the influence of the grain boundaries and is observed at lower frequencies.^25^ Taking into account the thickness of the pellet *L* and the area *A* of the electrodes, the conductivity was calculated according to σ = L × (*R*_electrolyte_ × *A*)^−1^. The activation energies (*E*_A_) were determined using the Arrhenius-type equation represented as σ*T* = σ_0_ exp­(−*E*_A_(*RT*)^−1^), which is derived from the Nernst–Einstein equation.^26,27^

Chronoamperometry measurements with blocking stainless steel electrodes were carried out to determine the ionic transference number (*t*_ionic_). The cell was polarized by applying an external voltage of 0.5 V and the current was measured over time. Both the electronic (*i*_electronic_) and ionic current (*i*_ionic_) contribute to the initially measured current (*i*_0_ = *i*_electronic_ + *i*_ionic_), whereas to the final current, only the electronic part is contributing (*i*_final_ = *i*_electronic_). The relevant value can be determined using the formula *t*_ion_ = (*i*_ionic_)­(*i*_initial_)^−1^.^10,28^ The lithium transport number (*t*_Li+_) was obtained in a symmetric cell configuration with nonblocking lithium metal electrodes. A small polarization voltage of 10 mV was applied, and the resulting current was measured over time. It can be assumed that the measured current after stabilization is mainly due to the migration of Li-ions. Hence *t*_Li+_ was estimated by dividing the steady state current (*I*_ss_) by the initial current (*I*_0_).^29^

For cyclic voltammetry (CV) measurements pellets with a diameter of 6 mm were used. For the anodic and cathodic stability measurements, 32.2 mg and 11.5 mg SSE were pelletized at 486 MPa, respectively. For the anodic CV measurement, a second layer was added to improve the electronic contact between the stainless steel electrode and the SSE by pressing 1.1 mg of a mixture (95:5 weight ratio) of the SSE and carbon (Ketjenblack EC600JD, Akzo Nobel Chemicals) with 486 MPa on top of the SSE pellet.^30^ A lithium disk prepared from a scratched and flattened lithium ribbon (99.9%, thickness 0.38 mm, Sigma-Aldrich) was used as a counter and reference electrode before the screws of the cell were tightened with a torque of 1.2 N m. The potential was varied from the open circuit voltage (OCV) to 4.5 V vs Li^+^/Li to probe anodic stability or from OCV to −0.1 V vs Li^+^/Li to probe the cathodic stability at a scanning rate of 100 μV s^–1^ after a resting period of 2 h. Cathode composite materials were obtained by either blending the SSE with TiS_2_ (99.9%, Sigma-Aldrich) in a 6:4 weight ratio or by mixing the SSE with LiFePO_4_ (Nanografi) in a 4:6 weight ratio by hand for 5 min. Multilayered pellets were prepared by cold-pressing 40/34 mg of SSE (0.6/0.5 mm thickness) uniaxially in a 6 mm pressing die and subsequently adding either 2.0 mg TiS_2_ composite cathode (0.676 mAh cm^–2^) or 1.2 mg LiFePO_4_-based cathode mixture (0.122 mAh cm^–2^) before pelletizing with 600 MPa. A lithium disk served as the negative electrode on the anode side. The as-prepared battery cells were closed with 3 screws with a torque of 1.2 N m and left to rest for 2 h before being cycled.

## 3 Results and Discussion

### 3.1 Structural Properties of Li_2_B_12_H_12_/ZrO_2_ Nanocomposites

The nanocomposites of Li_2_B_12_H_12_ and ZrO_2_ were prepared via ball milling. Figure 1a shows the XRD patterns of Li_2_B_12_H_12_/ZrO_2_ nanocomposites (with 25 vol % ZrO_2_) after various durations of mechanochemical treatment, as well as the pristine reference materials. A consistent composition of 25 vol % ZrO_2_ was utilized to ensure comparability, as this proportion has been shown to yield optimal performance in prior studies.^31,32^ Figure 1b displays an enlarged section with the main diffraction peaks of Li_2_B_12_H_12_. The diffraction pattern of the moderate temperature Li_2_B_12_H_12_ α-phase reflects the face-centered cubic unit cell (*Pa*3̅) with a peak maximum at 2θ = 18.45°.^33^ The shape of the main diffraction peak, as illustrated after 24 h of ball milling, changes and slightly shifts toward lower 2θ angles. This transformation of the peak shape corresponds to a partial β-Li_2_B_12_H_12_ phase formation (disordered enlarged fcc phase), accompanied by a shoulder indicating the copresence of the initial α-phase with decreasing intensity after 36 h of ball milling.^16^

**Figure 1:**
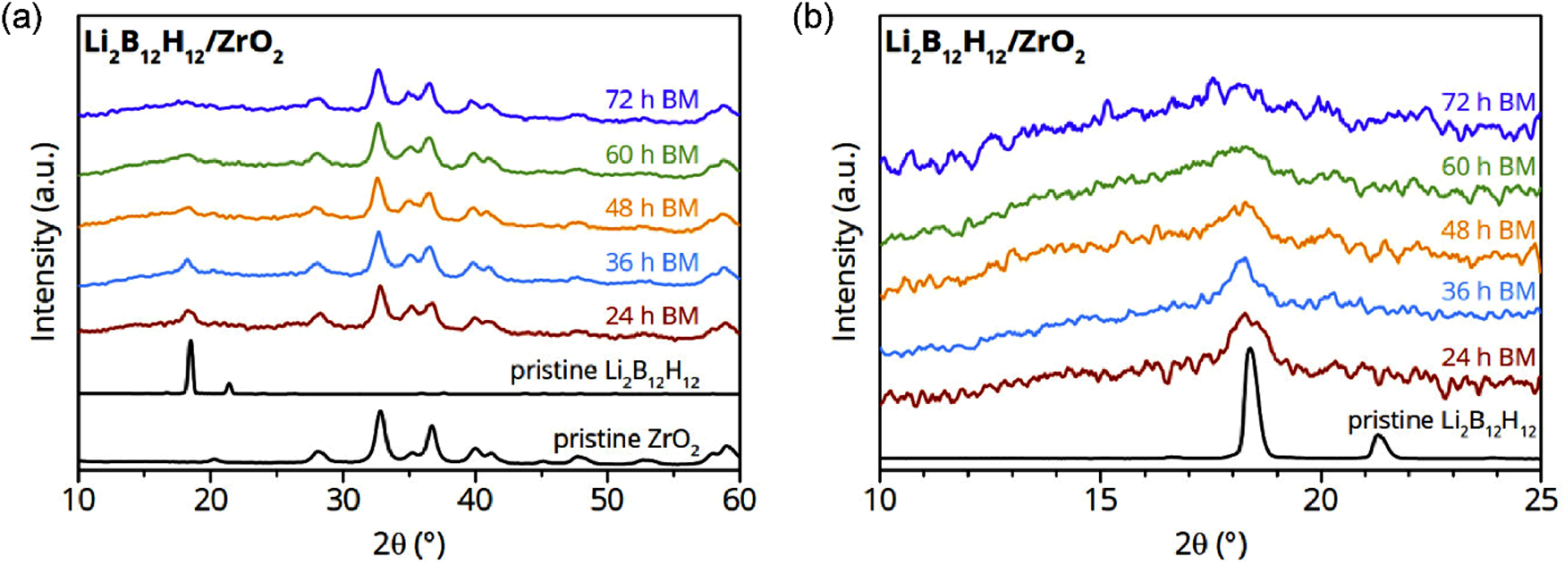
(a) XRD patterns after various durations of ball milling Li_2_B_12_H_12_ + 25 vol % ZrO_2_ in comparison with the pristine materials and (b) enlarged section between 2θ of 10° and 25°.

With increasing treatment time, all diffraction peaks corresponding to Li_2_B_12_H_12_ disappear gradually, leading to the absence of a recognizable peak after milling for 72 h. This suggests a reduction in crystallite size and eventually formation of an amorphous Li_2_B_12_H_12_ phase, or decomposition and reaction with ZrO_2_. However, the crystallinity of the ZrO_2_ scaffold did not change with increasing duration of ball milling, and no additional crystalline phase was observed even after prolonged ball milling. This shows that bulk chemical reaction with ZrO_2_ is not responsible for the disappearance of the Li_2_B_12_H_12_ diffraction peaks. Ball milling a mixture of Li_2_B_12_H_12_ and ZrO_2_ may result in atom-deficient complex (B_12_H_12_^2–^) anions due to hydrogen evolution at locally high temperatures (hot spots), which could also partially modify the ZrO_2_ surface.^12,34^ Furthermore, DSC analysis (Figure S2) revealed the absence of the polymorphic phase transition in the nanoconfined, amorphous Li_2_B_12_H_12_ samples, indicating that the nanocomposite formation with ZrO_2_ has indeed modified the Li_2_B_12_H_12_. Nevertheless, ICP results (Table S1) evidence that the nanocomposite has the expected elemental composition, indicating that the observed changes do not result from atmospheric moisture uptake. Additionally, negligible amounts of tungsten and carbon impurities were found, probably originating from the tungsten carbide grinding bowls and balls. However, as will be discussed later, the nanocomposite shows no electronic conductivity (Figure S5), thus, the negligible amounts of W and C impurities have no noticeable impact on the electrochemical properties.

To gain more information on the chemical state of the compound, the samples were analyzed by DRIFTS in argon atmosphere (moisture-free). The overview spectra of the pristine compounds and the nanocomposites are shown in Figure 2a. The spectrum of the pristine ZrO_2_ shows bands in the range 3504–3684 cm^–1^ and a broad band centered around ∼3311 cm^–1^, which are assigned to O–H stretching modes. Additional infrared active bands are observed in the range 1457–1558 cm^–1^, corresponding to the bending mode of the O–H surface groups.^35^ The spectrum of pristine Li_2_B_12_H_12_ shows three dominant overlapping peaks at ∼2500 cm^–1^ ascribed to B–H stretching vibrations, and a second feature at ∼1087 cm^–1^ could be observed, assigned to the combined motion of hydrogen and boron atoms against the valence stretching and bending force constant.^36,37^ After mechanochemical treatment, the B–H stretching mode is broadened, which reflects a change in the chemical environment such as distortion in the local symmetry.

**Figure 2:**
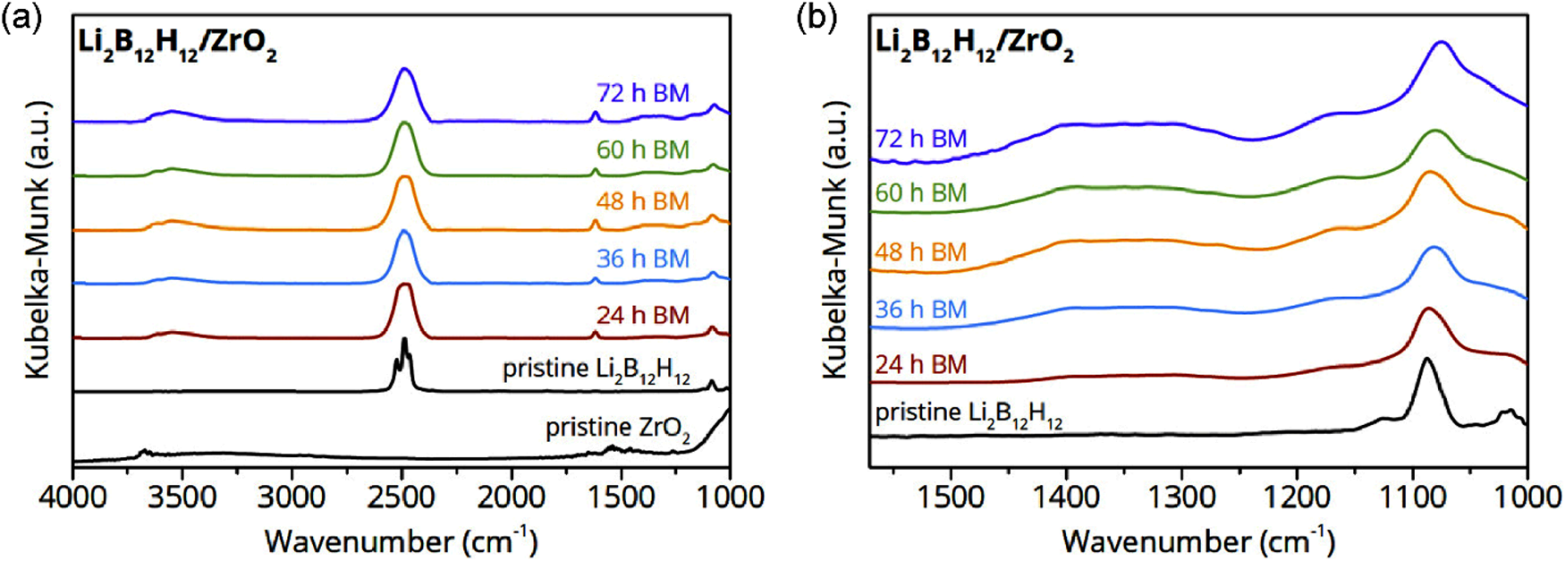
(a) DRIFTS data after various durations of ball milling Li_2_B_12_H_12_ + 25 vol % ZrO_2_ in comparison with the pristine materials. (b) Enlarged section of DRIFTS data displaying a peak evolution upon ball milling.

Interestingly, the bands corresponding to O–H surface groups of the pristine ZrO_2_ nanoparticles are absent in the nanoconfined samples, instead, several new features emerge between 3670–3330 cm^–1^ (Figure S3) and at ∼1615 cm^–1^. Moreover, the intensity increases with the duration of the mechanochemical treatment, suggesting that these bands result from an interaction between ZrO_2_ and Li_2_B_12_H_12_. This implies that the mechanochemical treatment leads to profound changes, especially in the stretching modes of the O–H groups on the ZrO_2_ surface. This could also explain the broadening of the dominant B–H stretching peak at ∼2500 cm^–1^. The disappearance of the oxide surface groups upon nanocomposite formation has been previously reported for nanocomposites of complex hydrides (such as LiBH_4_ and NaBH_4_) and oxides and was attributed to interface reaction with the complex hydrides.^38,39^ Beyond that, broad features, consisting of several overlapping peaks between 1100–1500 cm^–1^ emerged in the nanoconfined samples (Figure 2b). These bands are most likely associated with B–O stretching vibrations in trigonal BO_3_- and tetrahedral BO_4_-units, which could have formed during the mechanochemical treatment.^40,41^ However, the formation of the new vibrational peaks has not been reported before, suggesting that the interaction of ZrO_2_ with Li_2_B_12_H_12_ is different from the interaction with the aforementioned complex hydrides. This also indicates a significant change in the chemical bonding of Li_2_B_12_H_12_ upon nanocomposite formation with ZrO_2_.

### 3.2 Ionic Conductivity

The effect of the mechanochemical treatment on the conductivity of the nanocomposite made from Li_2_B_12_H_12_ and 25 vol % ZrO_2_ is shown in Figure 3a. The Arrhenius plot shows the conductivity versus the inverse temperature for the nanocomposite after 50 h ball milling and those of the pristine and ball-milled Li_2_B_12_H_12_. Remarkably, the conductivity of the synthesized composite is ∼1.2 × 10^–3^ S cm^–1^ at 60 °C, which is more than 3 orders of magnitude higher than that of the pristine compound and more than 10 times higher than that of the ball-milled Li_2_B_12_H_12_. Note that the absolute conductivity depends on the exact preparation conditions, especially the ball milling time (Figure 3b) and the ball-to-sample ratio are important factors influencing the overall result. Generally, the highest conductivity is obtained after ∼50 h of ball milling and does not change significantly if the mechanochemical treatment time is extended. A representative Nyquist plot is presented in Figure S4, together with the equivalent circuit model used to fit the data.

**Figure 3:**
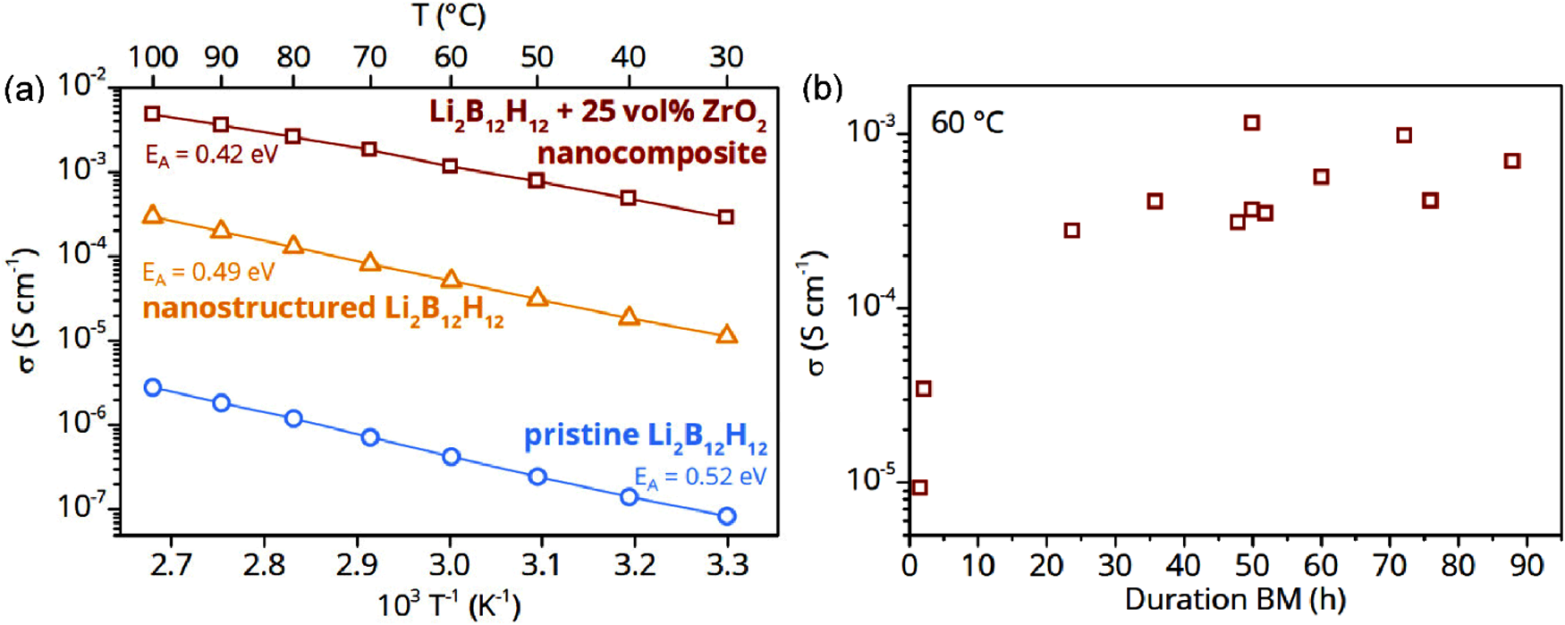
(a) Ionic conductivity of the nanocomposite containing Li_2_B_12_H_12_ + 25 vol % ZrO_2_ after 50 h ball milling in comparison to pristine and mechanochemical treated Li_2_B_12_H_12_ material as a function of temperature. (b) Li-ion conductivity at 60 °C of a nanocomposite containing 25 vol % ZrO_2_ as a function of the ball milling duration.

The linear increase in conductivity with rising temperatures for all three, the pristine Li_2_B_12_H_12_, the nanostructured (ball milled) Li_2_B_12_H_12_ and the nanoconfined samples indicate that the conduction mechanism is based on a thermally activated process (ion jump) and that the samples are stable in the measured temperature range. It is noteworthy that the activation energy differs, with 0.42 ± 0.01 eV for the 50 h mechanochemically treated SSE from 0.52 ± 0.01 eV for the pristine complex metal hydride. This suggests that nanocomposite formation modified and enhanced the ion conduction mechanism of Li_2_B_12_H_12_. The enhanced conductivity and lower activation energy for the nanocomposite could be due to the formation of a space-charge layer, or highly conductive interphase at the ZrO_2_–Li_2_B_12_H_12_ interface, which has been reported in some nanocomposite solid electrolytes based on complex hydrides.^16,20,42^ Alternatively, Li_2_B_12_H_12_ is a good reducing agent and, hence, can partially reduce ZrO_2_ leading to electronic conductivity. To investigate this, we determined the ionic transfer number using chronoamperometry. By taking into account the instrument noise, *t*_ionic_ is unity (1), showing that the conductivity of Li_2_B_12_H_12_ + 25 vol % ZrO_2_ is purely ionic (Figure S5a). In addition, the lithium transport number was estimated to be *t*_Li+_ > 0.9, which shows that the charge transport in the corresponding sample is overwhelmingly due to the movement of Li-ions (Figure S5b).

### 3.3 On the Origin of Fast Ionic Conduction in Li_2_B_12_H_12_ Nanocomposites

In line with the structural and chemical changes in the nanocomposites revealed by the DRIFTS measurements, interphase formation was considered a major possible cause of the enhanced ionic conductivity and electrochemical properties. Therefore, STEM coupled with EELS was used to investigate the interface of the complex metal hydride and the ZrO_2_ scaffold with spatial resolution. To exclude a misinterpretation of the Zr M_4/5_-edge signal (*E*_loss_ = 180/182 eV) in the vicinity of the B K-edge signal (*E*_loss_ = 188 eV), the Zr distribution was mapped by EDX spectroscopy. The elemental distribution of Li, B, and Zr in Figure 4 shows B-enriched areas, clearly distinguishable from areas containing higher amounts of Li and Zr, even though nanoscale proximity is maintained. The inverse correlation between B and Li is additionally illustrated in the relative elemental distribution map in Figure S6. The separation between B and Zr evidence that Li_2_B_12_H_12_ is nanoconfined in the interparticle pores of the ZrO_2_ scaffold. Intriguingly, a detailed look at the EDX elemental map shows partial enrichment of Li around the Zr at the interface region, which could indicate an interaction between Li-ions and oxygen vacancies with a high electron density in the ZrO_2_.^43^ Although the closeness of the Li K-edge (55 eV) with the broad Zr N_1_-edge (51 eV), might contribute to this observation, no clear signal from Zr N_1_-edge could be observed in the EELS data from this region (Figure S7). Thus, the STEM-EELS elemental distribution maps strongly support the DRIFTS results by indicating a modification in the chemical nature of the Li_2_B_12_H_12_ at the interface with ZrO_2_. An interphase layer was formed, which is suspected to be the origin of the fast ionic conduction.^20^

**Figure 4:**
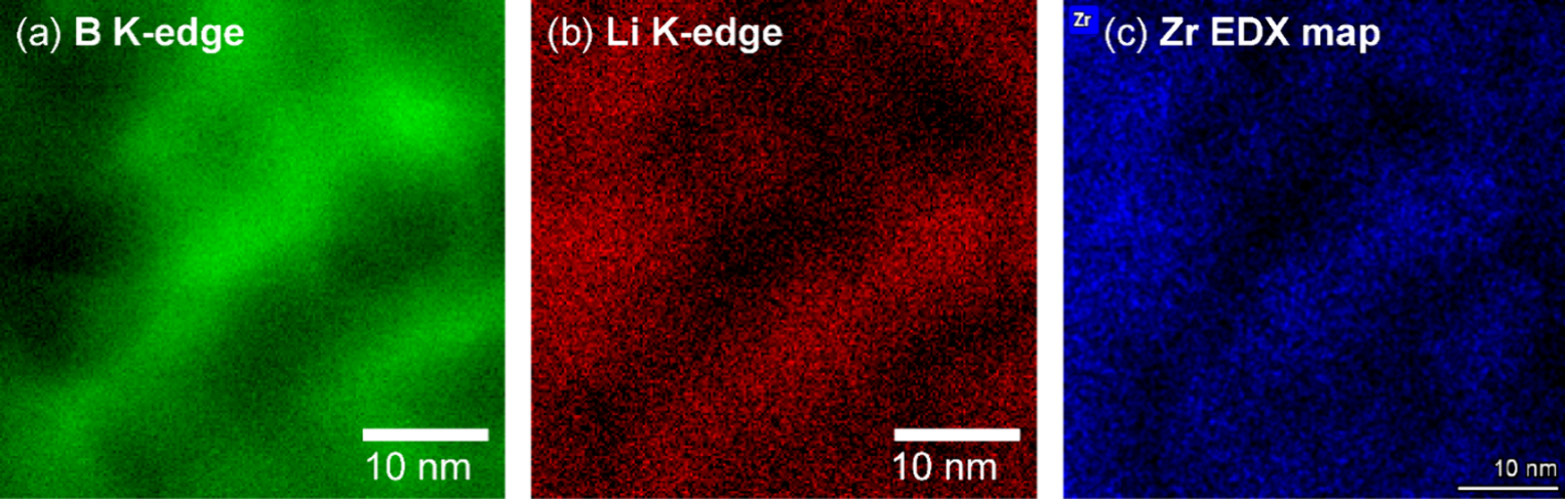
(a) Boron and (b) lithium elemental distribution mapped with electron energy loss spectroscopy and (c) zirconium elemental distribution mapped with energy-dispersive X-ray spectroscopy of the nanocomposite containing Li_2_B_12_H_12_ + 25 vol % ZrO_2_ after 50 h ball milling.

We also performed synchrotron based XRS measurements to gain a detailed insight into the change in the electronic structure of the materials in the nanocomposites, i.e. the composition of the interphase layer. The samples with 50 and 75 vol % ZrO_2_ contain a high fraction of insulating oxide (83.3 and 93.8 wt %, respectively) and therefore exhibit low ionic conductivities. Nevertheless, these nanocomposites, which contain less Li_2_B_12_H_12_, were studied in order to enhance the contribution of the interfacial interaction and exclude the contribution of bulk-like material.^20^

Due to the high concentration of ZrO_2_ in the nanocomposites, the contribution from the Zr M_4,5_-edge, becomes dominant and coincides with the first B K-edge peak I of Li_2_B_12_H_12_ at *E*_loss_ = ∼191.4 eV (Figure S8). Therefore, Figure 5a shows background corrected spectra in the region of the B K-edge, with the contribution of the partly overlapping Zr M_4,5_-edge subtracted. A peak at ∼191.3 eV has been observed before for tetrahedrally shaped BH_4_^–^ and the icosahedral shaped CB_11_H_12_^–^ and B_12_H_12_^2–^ anions based on the spectra of LiBH_4_, NaBH_4_, NaCB_11_H_12_ and MgB_12_H_12_ reported by de Kort et al.,^20^ Gulino et al.^44^ and Sahle et al.^45^ Note that the closo-borate anion consists of 12 B atoms in an icosahedral moiety, connected via nonclassical bonds as first described by Wade’s rules.^46^ Peak I is therefore associated with the transition of a B 1s electron from the closo-borate anion into an unoccupied boron antibonding π* (2a_1_) orbital.^20,44^

**Figure 5:**
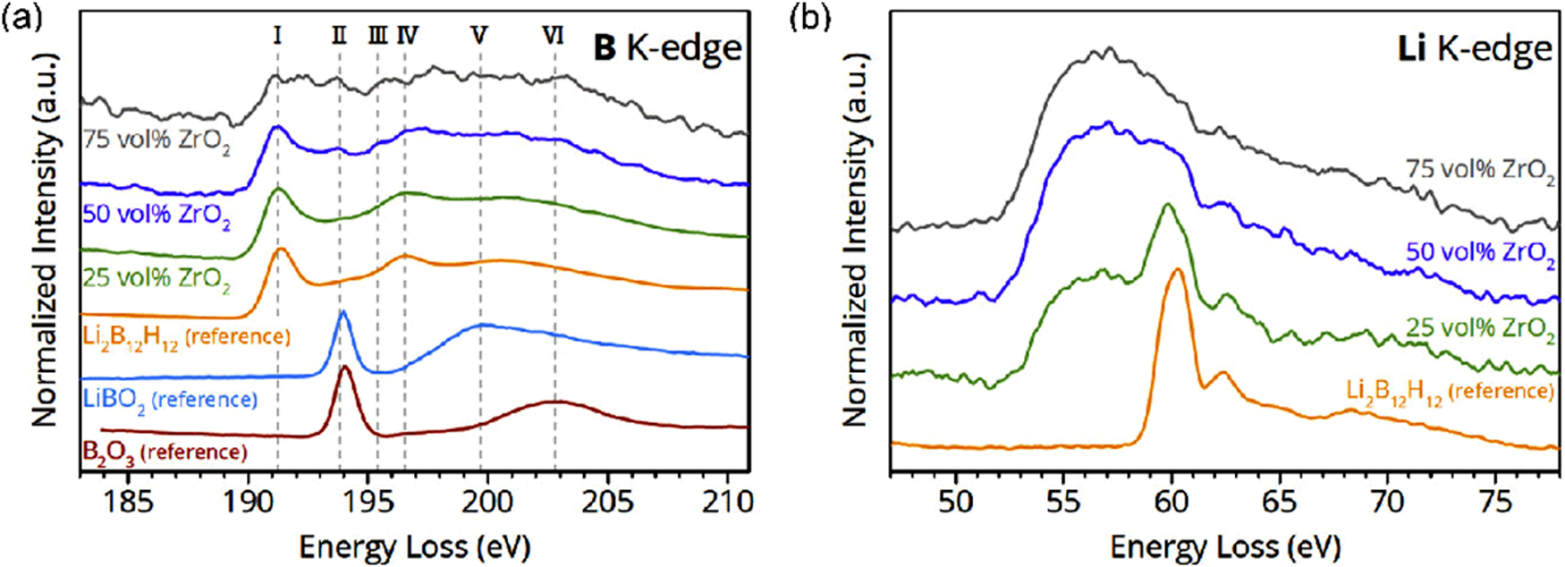
XRS spectra of reference compounds and different Li_2_B_12_H_12_ + ZrO_2_ nanocomposites, corrected for the Zr M_4,5_-edge contribution in the (a) boron K-edge region, and (b) Li K-edge region.

Furthermore, peak II at *E*_loss_ = ∼193.9 eV and the shoulder III at *E*_loss_ = ∼195.4 eV appear in the nanocomposite samples and become more dominant with decreasing Li_2_B_12_H_12_ (or increasing scaffold) concentration. Based on our approach, we can assign peaks II and III to the interphase layer, and by comparison with the B_2_O_3_ and LiBO_2_ reference samples, it is evident that II depicts B–O bonding characteristics. In trigonal planar B-containing compounds like B_2_O_3_, LiBO_2_ or BF_3_ peaks in this region are assigned to the B electronic transition 1s → 2p_*z*_ ^47–49^ This suggests that the nature of the interphase layer involves a chemically altered B_12_H_12_^2–^ anion, exhibiting a trigonal B–O bond character. This could be related to its interaction with ZrO_2_ surface hydroxyl groups.^48,50^

Moreover, the feature IV of pristine Li_2_B_12_H_12_ at *E*_loss_ = ∼196.6 eV fades with increasing ZrO_2_ content. This feature is related to the transition of B 1s electron into an unoccupied σ* orbital of t_2_ symmetry in icosahedral or tetrahedral coordinated boron.^44,51^ In contrast, the plateau region in the nanocomposite samples between IV and VI is slightly extended toward higher energy loss and the shape is more flattened compared to pristine Li_2_B_12_H_12_. This is likely because of the growing influence of the broad features V and VI originating from LiBO_2_ and B_2_O_3_-like compounds, representing various transitions from B 1s electrons into empty and antibonding B–O orbitals.^47^ All of the observed changes in the B electronic structure support the hypothesis that the chemical nature of B at the interface between ZrO_2_ and Li_2_B_12_H_12_ is quite distinct from the bulk compound.

We also examined the electronic structure of lithium for which the corresponding spectra are presented in Figure 5b. Due to the overlap of the Li K-edge with the broad Zr N_1_-edge feature centered around *E*_loss_ = ∼56 eV, interpretation of the results is challenging. Additionally, the unavailability of reference data prevents the isolation of the Li K-edge signal from the Zr N_1_-edge contribution. Moreover, unlike in the case of STEM-EELS with a high spatially resolved spectrum, allowing mapping of different regions at the interphase, the contribution from the Zr N_1_-edge cannot be excluded in the XRS spectra due to the bulk nature of the technique in this respect. The spectrum of pristine Li_2_B_12_H_12_ displays an intense peak at ∼60.3 eV, accompanied by a secondary feature between 62–65 eV. In the spectrum of the nanocomposite containing 25 vol % ZrO_2_, the maximum of the intense peak is shifted toward lower energy loss ∼59.8 eV. However, in the samples containing 50 or 75 vol % ZrO_2,_ the shift is less evident because of the overlapping Zr N_1_-edge. A possible explanation could be the interaction of the Li-ions with the oxygen defects (vacancies and interstitials) at the interface with the ZrO_2_ scaffold material, as also suggested by the STEM-EELS results. Such interaction will result in a peak at lower energy loss, as previously observed in lithium halides with decreasing electronegativity difference.^47^ Furthermore, the formation of “B–O” like bond at the interface will inadvertently influence the chemical environment of Li due to the altered interaction with the B_12_H_12_^2–^ anion.

These findings, combined with the partial spatial segregation of Li and B revealed by the STEM-EELS-EDX results, and the disappearance of the hydroxyl vibrational mode of the ZrO_2_ scaffold (DRIFTS), firmly suggest a strong interface interaction/reaction between Li_2_B_12_H_12_ and the surface groups of ZrO_2_ scaffold. Although the exact chemical composition of the highly defective and disordered interphase remains unclear, the resulting high ionic conductivity can be attributed to the aforementioned interface interaction.

### 3.4 Electrochemical Properties and the All-Solid-State Battery Test

Next to the high ionic conductivity, a wide electrochemical stability window is important to ensure compatibility of the nanocomposite electrolytes with high voltage cathode materials and allow the assembly of high energy density batteries. To identify the oxidative limit of the nanocomposites SSE we measured CV at 60 °C (Figure 6a). The SSE was blended with electrically conductive carbon to increase the contact area with the working electrode and thereby increase the decomposition kinetics to facilitate signal detection.^52,53^ An irreversible oxidative event with an onset voltage of *E*_onset_ = 3.8 V vs Li^+^/Li was obtained by linear regression of the anodic current in the first CV cycle.^30^ No further anodic or cathodic currents could be observed during the subsequent cycles, suggesting that the observed oxidative event in the first cycle results in stable solid electrolyte interphase (SEI) formation or some irreversible electrochemical reaction. The cathodic stability of the nanocomposite was assessed from OCV to −0.1 V vs Li^+^/Li (Figure S9). Apart from lithium plating and stripping around 0 V vs Li^+^/Li, no reductive or oxidative processes were detected. The Nyquist plot in Figure 6b compares EIS data obtained before and after the CV measurement and shows that the interface resistance is slightly increased and the shape of the Warburg element is changed, due to the electrochemically induced modification of the interface between the SSE and the stainless steel electrode. The overall oxidative stability of the nanocomposite is improved by comparison with the value of 3.6 V vs Li^+^/Li at 120 °C for the pristine Li_2_B_12_H_12_ previously reported by Garcia et al.^54^ Nevertheless, higher temperatures could also be responsible for the accelerated decomposition. It is worth mentioning that the formation of a stable SEI and cathode electrolyte interphase is essential and can significantly improve the cycling stability of electrolytes by preventing further decomposition.

**Figure 6:**
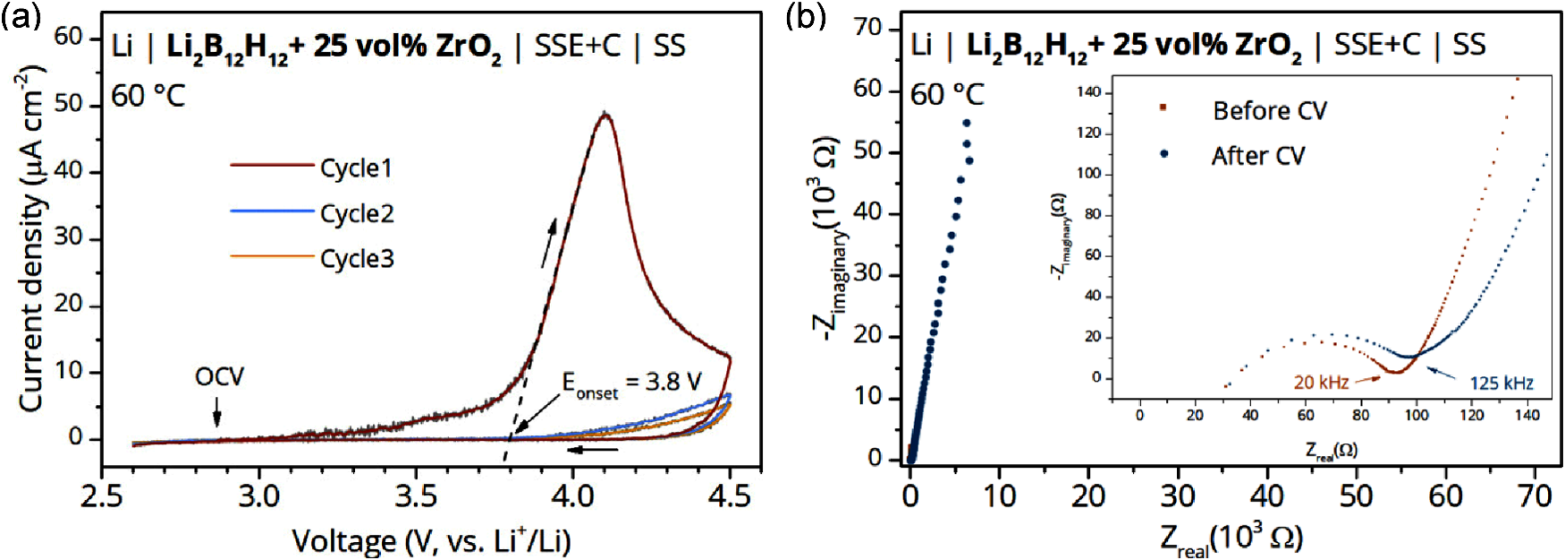
(a) Cyclic voltammetry curve of the SSE (Li_2_B_12_H_12_ + 25 vol % ZrO_2_) measured with a scan rate of 100 μV s^–1^ from OCV to 4.5 to 2.6 V vs Li^+^/Li at 60 °C. *E*_onset_ was estimated by linear regression of the anodic current. (b) Nyquist plot of the EIS data obtained before and after the CV measurement, with enlarged inset.

Given the high ionic conductivity and electrochemical stability of the nanocomposites, we investigated its properties in an ASSB composed of Li metal anode and TiS_2_ cathode composite, applying a stack pressure of 106 MPa while closing the cell. The galvanostatic cycling behavior of the ASSB at 60 °C, and at different C-rates is shown in Figure 7. Clearly, the nanocomposite SSE is compatible with a Li metal and TiS_2_ and demonstrates good stability over 170 charge–discharge cycles. The cathode material TiS_2_ was chosen because the working voltage of about 2.0 V vs Li^+^/Li falls within the electrochemical stability window of the SSE and is feasible for use in composite cathodes due to its electrical conductivity.^55^ The utilization of Li metal in batteries contributes to a higher energy density than intercalation anodes but also requires the SSE to prevent dendrite formation and mechanical resistance due to volume changes during cycling. Constant current charging and discharging at different C-rates with potential limits of 1.7 and 2.5 V was carried out at 40–60 °C to ensure sufficient ionic conductivity, which is required for cycling the battery at higher currents.

**Figure 7:**
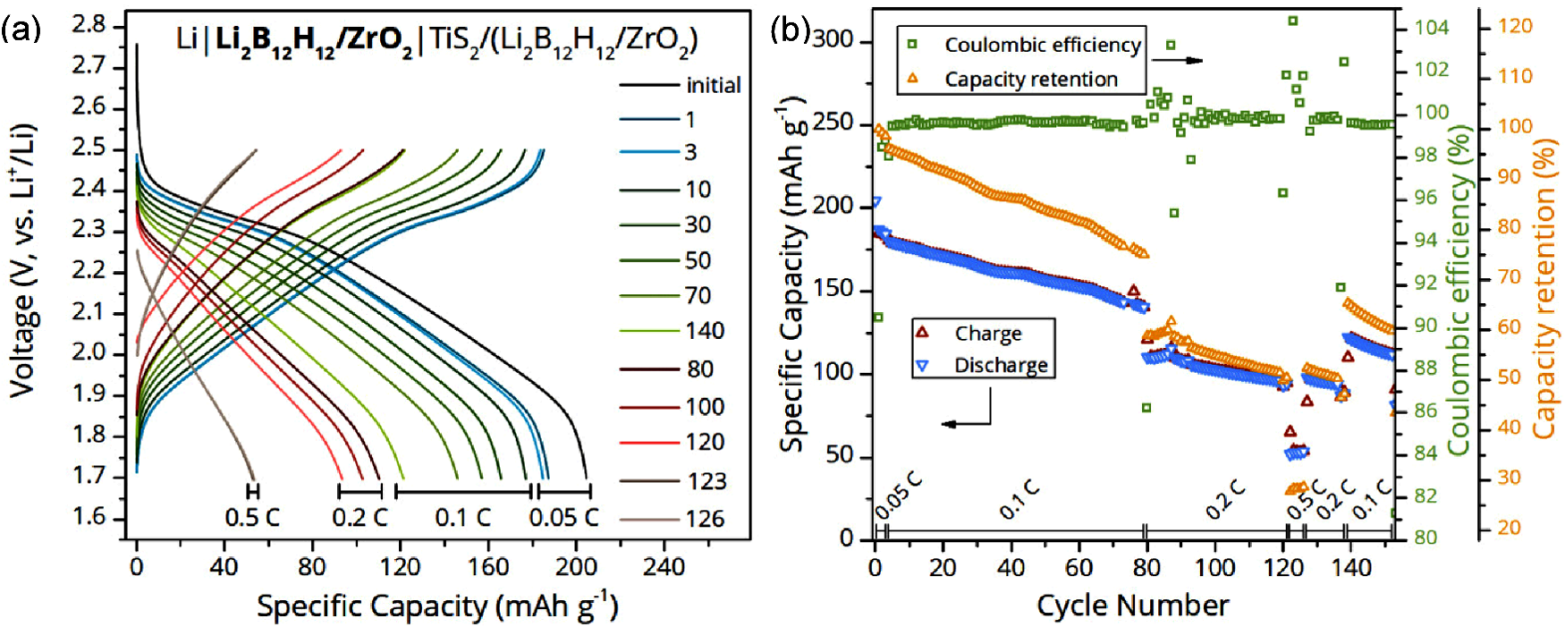
Battery performance of a Li|Li_2_B_12_H_12_ + 25 vol % ZrO_2_|TiS_2_/(Li_2_B_12_H_12_ + 25 vol % ZrO_2_) cell, cycled at 60 °C and various C-rates. (a) Selected voltage profiles and (b) charge/discharge specific capacity, Coulombic efficiency and capacity retention as a function of cycle number.

Before the initial discharge at C/20, the battery was allowed to rest for 2 h to adjust to the ambient temperature. At the end of the initial discharge, a specific capacity of 205 mAh g^–1^ could be measured, which is close to the theoretical value of ∼239 mAh g^–1^.^55–57^ Following three initial cycles at C/20 (33.8 μA cm^–2^), the battery underwent 70 charging and discharging cycles at C/10 (67.6 μA cm^–2^) followed by a series of faster charging rates of C/5 (0.14 mA cm^–2^) and C/2 (0.34 mA cm^–2^) before being operated again at C/10. This protocol demonstrates that higher capacity at lower C-rates can be achieved reversibly (Figure 7b). A continuous capacity loss of approximately 0.26% per cycle was measured, corresponding to 60.5% capacity retention after 150 cycles. This is reflected in the increased cell impedance, displayed in Figure S10. Among the formation of SEI and CEI, an increasing contact resistance with lithium metal and the cathode material TiS_2_, caused by the volume expansion of about 8.8% during lithiation, are considered responsible for the capacity loss.^5,58,59^ In general, the Coulombic efficiency showed stable values averaged to 99.7%, except for the cycles after a change in the current regime, which led to a shortfall or surplus depending on higher or lower applied current densities. Further investigation is aimed at understanding the effects of temperature on the performance of the battery. Figure S11 shows that the specific capacity of the battery decreases when the cycling temperature is reduced. This reduction in capacity is attributed to the higher ohmic resistance within the battery cell, due to lower ionic conductivity at lower temperatures, which impedes efficient ion transport and overall performance.

We also explored the suitability of Li_2_B_12_H_12_/ZrO_2_ in combination with a LiFePO_4_ cathode composite and lithium metal anode at an assembly stack pressure of 106 MPa. The cycling behavior of the LiFePO_4_-based ASSB is presented and discussed in the Supporting Information (Figures S12 and S13). Although the cell is not yet fully optimized, the battery exhibited interesting performance, and scanning electron microscopy of the cycled battery pellet confirmed the absence of dendritic Li growth after 9 + 34 cycles at C/10 (43.3 μA cm^–2^) and C/5 (86.6 μA cm^–2^) at 60 °C as showed in Figure S12. This demonstrate that Li_2_B_12_H_12_/ZrO_2_ nanocomposites are promising SSEs for ASSBs, and that interface engineering is a versatile strategy for further improvement of the electrochemical properties and performance.

## 4 Conclusions

We have investigated the effects of nanocomposite formation on the Li-ion conductivity and the electrochemical properties of a Li_2_B_12_H_12_ based solid electrolytes in all-solid-state batteries consisting of Li metal anode and either TiS_2_ or LiFePO_4_ cathode. Li_2_B_12_H_12_/ZrO_2_ nanocomposites were successfully prepared by mechanochemical treatment of Li_2_B_12_H_12_ + 25 vol % ZrO_2_ mixture. This resulted in a remarkable (3 orders of magnitude) increase in the Li-ion conductivity. A systematic investigation of the nanocomposite solid electrolyte as a function of ball milling duration indicates lattice distortion and reduction in crystallite sizes, resulting in an amorphous Li_2_B_12_H_12_ phase in the nanocomposite. Electron microscopy coupled with electron energy loss spectroscopy and energy dispersive X-ray spectroscopy confirmed nanoscale proximity between Li, B and Zr. In addition, a partial enrichment of Li in intimate contact with the oxidic scaffold was observed, demonstrating the formation of a highly modified conductor–insulator interface. Both X-ray Raman scattering and infrared spectroscopy measurements revealed the formation of an interphase with an altered electronic structure and vibrational modes with a B–O like bond character. These results show that the conductivity enhancement stems from the formation of a highly defective and conductive interphase. Finally, we demonstrate that the synthesized nanocomposite material is compatible with lithium metal and can inhibit the growth of dendrites in an all-solid-state battery (Li|Li_2_B_12_H_12_/ZrO_2_|LiFePO_4_). This manifests the potential of nanocomposite formation/interface engineering to improve the electrochemical properties of solid electrolytes.

## Supplementary Material



## Data Availability

The data underlying this study are openly available in Zenodo data repository at 10.5281/zenodo.14040588.

## Notes

Figure S1: electrochemical cell schematic; Figure S2: DSC curves; Table S1: elemental analysis; Figure S3: DRIFTS data; Figure S4: Nyquist plot SSE; Figure S5: chronoamperometry measurements; Figure S6: relative STEM-EELS map; Figure S7: ADF-STEM image with different regions of interest and raw EELS data; Figure S8: XRS spectra; Figure S9: cyclic voltammetry; Figure S10: Nyquist plot battery cell; Figure S11: battery cycling data (TiS_2_ cathode); Figure S12: SEM images; and Figure S13: battery cycling data (LiFePO_4_ cathode) (PDF)
