# Supplementary material for: Enhanced Ionic Conductivity and Electrochemical Properties of Li2B12H12/ZrO2 Nanocomposites for All-Solid-State Lithium Metal Batteries
Source: ACS Appl Mater Interfaces. 2025 Jun 2;17(23):33824–33. doi: 10.1021/acsami.5c01939 (PMC12163931; doi:10.1021/acsami.5c01939)
Supplement: Supplementary file 1 [file am5c01939_si_001.pdf]

Supporting Information

Enhanced Ionic Conductivity and Electrochemical Properties of $Li_2B_{12}H_{12}/ZrO_2$ Nanocomposites for All-Solid-State Lithium Metal Batteries

Jonas D. Hehn[a], Hendrik P. Rodenburg[a], Masoud Lazemi[a], Juliette C. Verschoor[a], Marta Perxés Perich[a], Martin Sundermann[b,c], Hlynur Gretarsson[b], Jessi E. S. van der Hoeven[a], Frank M. F. de Groot[a], Petra E. de Jongh[a] and Peter Ngene[a]*

[a] *Materials Chemistry and Catalysis, Debye Institute for Nanomaterials Science, Utrecht University, Utrecht 3584 CG, The Netherlands*

[b] *Deutsches Elektronen-Synchrotron DESY, 22607 Hamburg, Germany*

[c] *Max Planck Institute for Chemical Physics of Solids, 01187 Dresden, Germany*

* Corresponding author Email: p.ngene@uu.nl

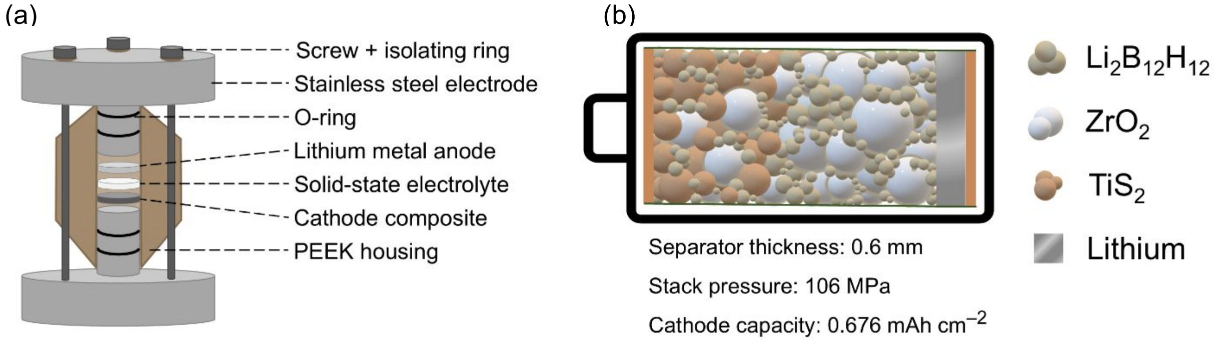

Figure S1. (a) Schematic drawing of the electrochemical cell and (b) schematic of the cell composition with key parameters.

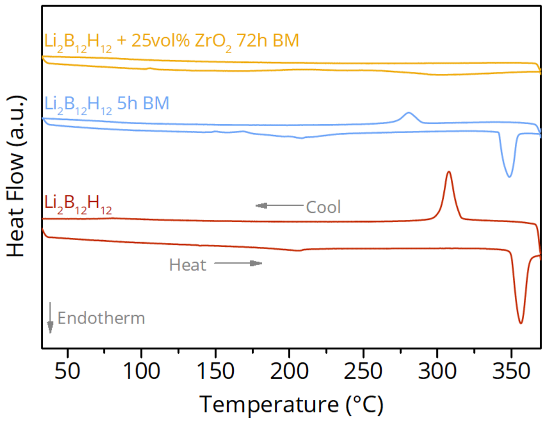

Figure S2. DSC curves of $Li_2B_{12}H_{12}$ + 25 vol% ZrO_2 in comparison with pristine and ball milled $Li_2B_{12}H_{12}$.

DSC analysis for pristine $Li_2B_{12}H_{12}$ revealed an endothermic event at ~356 °C, corresponding to the polymorphic phase transition, while the mechanochemically treated compound undergoes the phase transition at slightly lower temperatures. In contrast, the solid-solid phase transition is absent in the nanoconfined sample.

Table S1: Results of the elemental analysis with inductively coupled plasma of $Li_2B_{12}H_{12}$ + 25 vol% ZrO_2 after 50 h of ball milling.

	**Lithium**	**Boron**	**Hydrogen**	**Zirconium**	**Tungsten**	**Carbon**
**Mass (wt%)**	3.25	30.41	2.84	44.91	0.29	0.41
**n (mol)**	2.00	12.00	12.02	2.10	0.01	0.15

Elemental analysis was used to quantify the elements in the $Li_2B_{12}H_{12}$ + 25 vol% ZrO_2 nanocomposite. The results show that the sample has the expected elemental composition. Interestingly, there is a slim excess of hydrogen (12.02 mol instead of 12.00 mol). This is likely from the surface hydroxyl group of the ZrO_2, which contribute to the measurement.

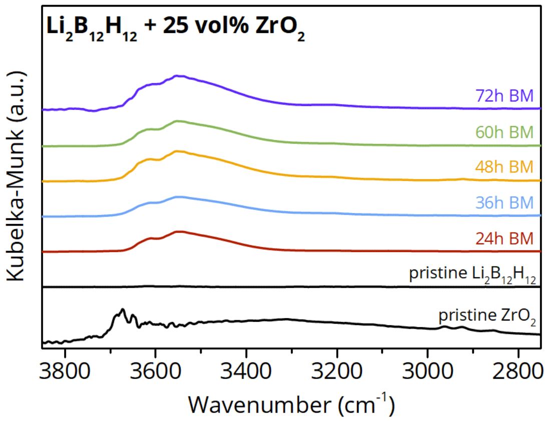

Figure S3. Enlarged section DRIFTS data after various durations of ball milling $Li_2B_{12}H_{12}$ + 25 vol% ZrO_2 in comparison with the pristine materials.

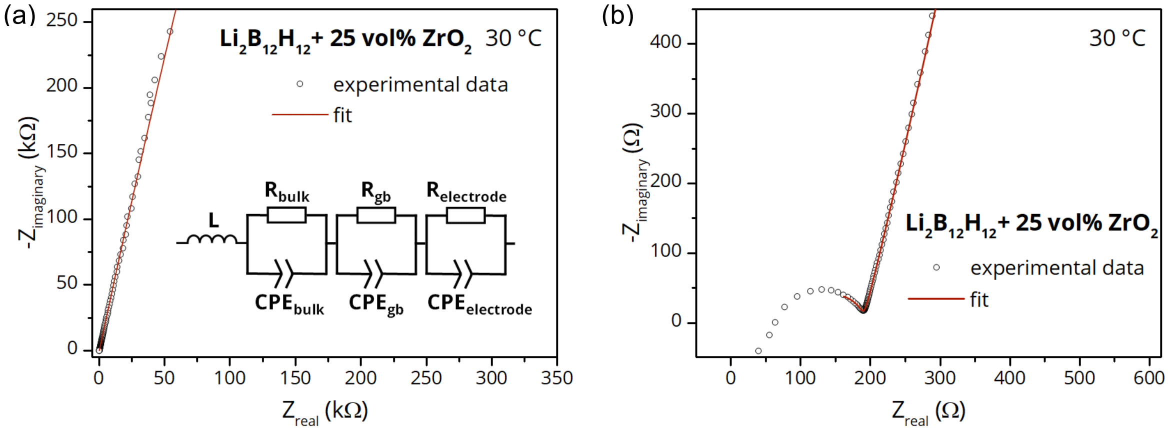

Figure S4. Nyquist plot covering the (a) full frequency range or (b) high frequency region of the obtained EIS data for $Li_2B_{12}H_{12}$ + 25 vol% ZrO_2 after 50 h ball milling. The inset in (a) shows the equivalent circuit model used for fitting.

The equivalent circuit model used for fitting the EIS data is shown as inset in **Figure S4**a in addition to the observed experimental data and fit for $Li_2B_{12}H_{12}$ + 25 vol% ZrO_2 after 50 h ball milling. To make up for inductance of the cables and setup an inductor was used in the equivalent circuit model. Two sets of a resistor *R* and a constant phase element *CPE* in parallel are connected in series and were used to account for the bulk and grain boundary resistivity of the sample. The contribution of the stainless steel blocking electrodes was included by a third (*R-CPE*) unit connected in series.

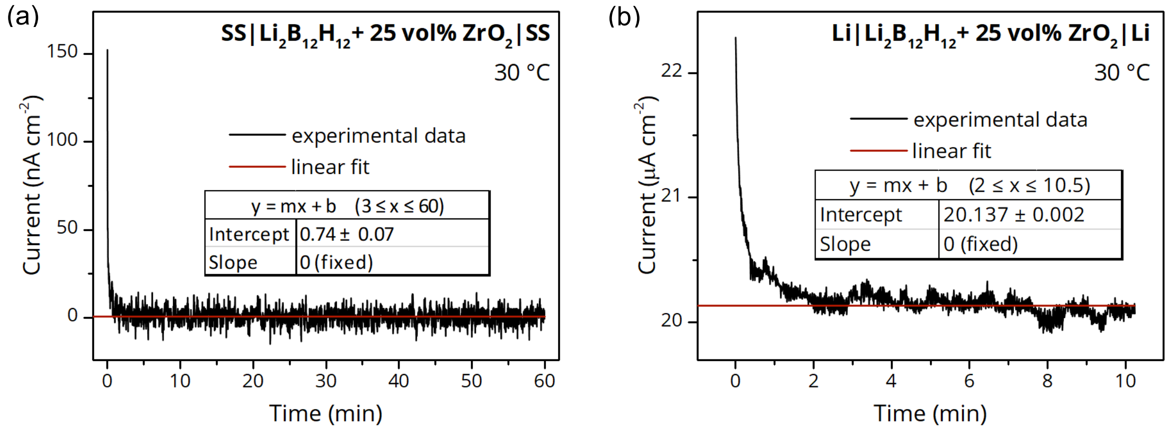

Figure S5. (a) Chronoamperometry measurement (U = 0.5 V) for $Li_2B_{12}H_{12}$ + 25 vol% ZrO_2 after 88 h ball milling with blocking stainless steel electrodes. The mean value for the experimental data x ≥ 3 min is approximately 1.52 nA/cm^2 and is regarded as instrumental noise (b) Chronoamperometry measurement (U = 10 mV) for $Li_2B_{12}H_{12}$ + 25 vol% ZrO_2 after 88 h ball milling with non-blocking lithium metal electrodes. The current stabilized at approximately 20.137 µA cm^{-2} for x ≥ 2 min.

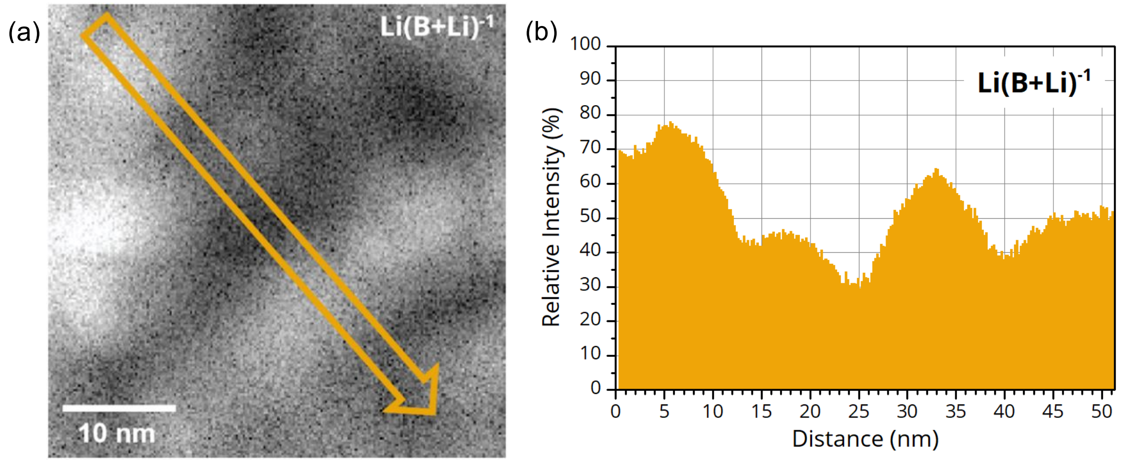

Figure S6. (a) Relative Li(B+Li)$^{-1}$ elemental distribution map. The arrow indicates the direction of the line scan (b).

A spectrum image of 200x198 pixels with a pixel size of 0.2 nm and 2 ms per pixel was acquired. Low-loss spectra were acquired with an exposure of 48.8 µs over a range of −30 eV to 277.2 eV and high-loss spectra with a pixel time of 1.95 ms over a range of 45 eV to 352.2 eV. The data was processed using the Gatan Microscopy Suite software. First, the low-loss spectra were used to align the zero-loss peak at each pixel, using the align SI by peak function in the software. The correction was applied to the high-loss spectra. A power-law curve was used for background subtraction. The Li K-edge (55 eV) and the B K-edge (188 eV) were tracked by subtraction of the background and mapping the signal from 55.4 eV to 94.2 eV and 187.1 eV to 234.9 eV, respectively. The Zr $M_{4,5}$-edge (180/182 eV) was not visible in the spectra. The relative elemental distribution map was generated by normalizing the lithium and boron EELS signal from 0 to 1 and then dividing the lithium signal by the combined signals of boron and lithium.

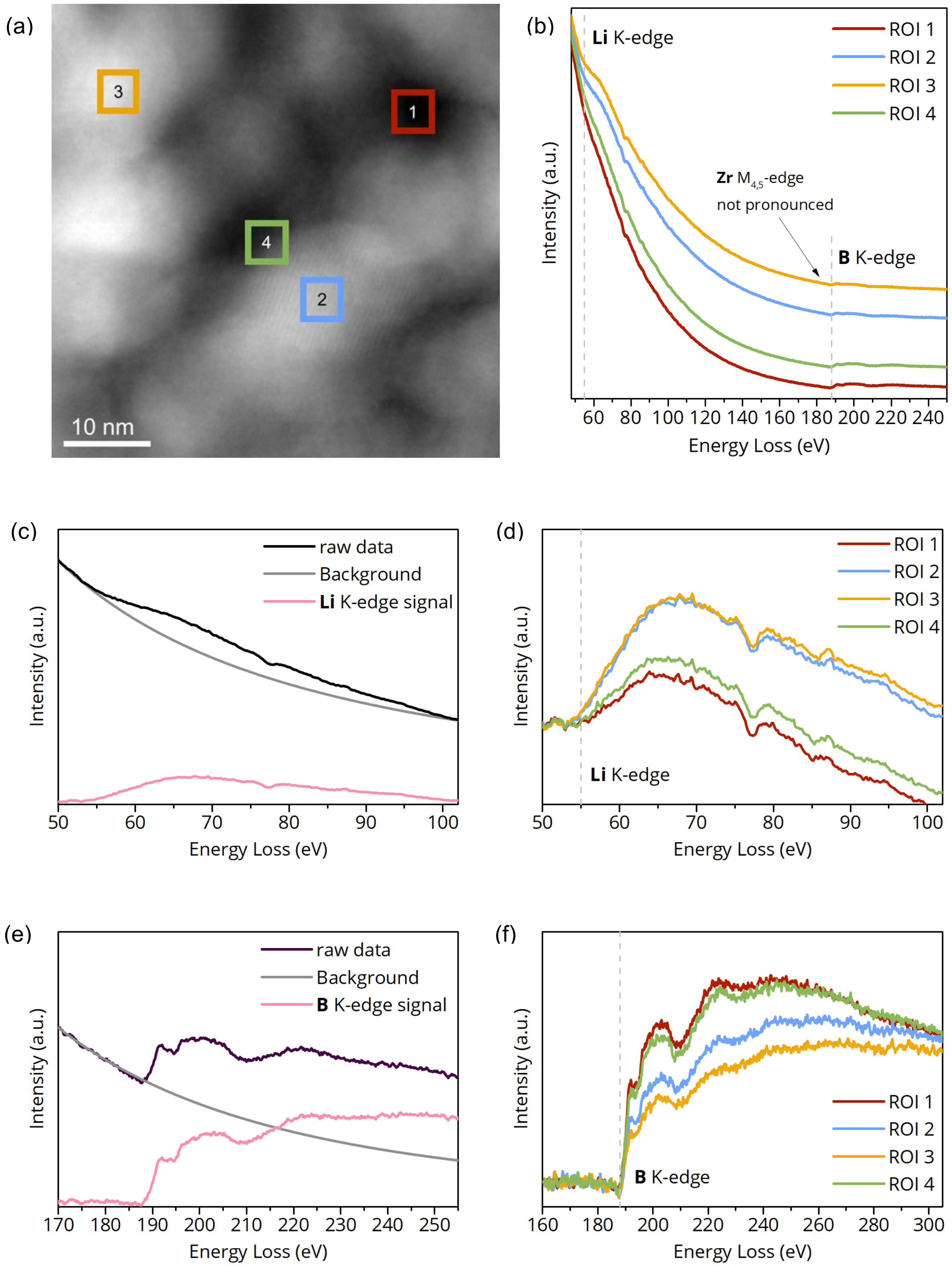

Figure S7. (a) ADF-STEM image with different regions of interest (ROI). Corresponding spectra are representing the raw electron energy loss data (b) and examples of the background removal and the background corrected spectra of the Li K-edge (c, d) and the B K-edge (e, f).

The EELS data neither show a signal for the minor Zr N_1-edge (51 eV) nor the major Zr $M_{4,5}$-edge (180/182 eV). Therefore, the signal in the corresponding regions was completely assigned to either the Li K-edge or the B K-edge.

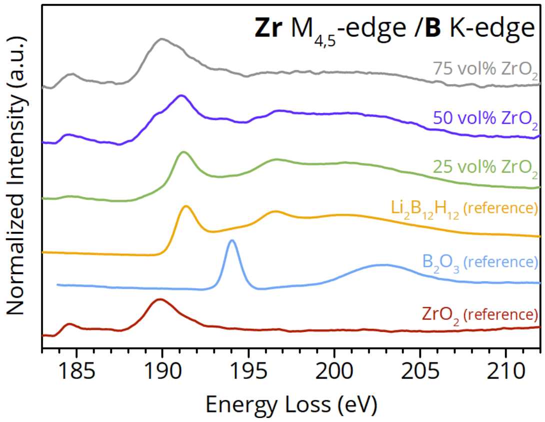

Figure S8. XRS spectra of reference compounds and different $Li_2B_{12}H_{12}$ + ZrO_2 nanocomposites in the Boron K-edge and Zr $M_{4,5}$-edge region.

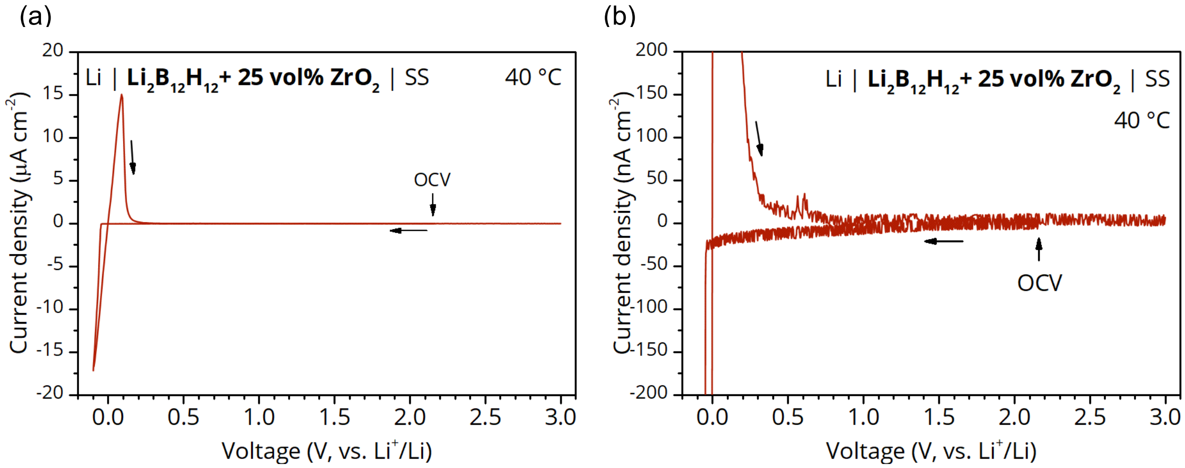

Figure S9. Cyclic voltammetry measurement (a) overview and (b) zoomed in view of the SSE ($Li_2B_{12}H_{12}$ + 25 vol% ZrO_2) recorded with a scan rate of 100 μV s^{-1} from OCV to -0.1 V to 3 V *vs.* Li^+/Li at 40 °C.

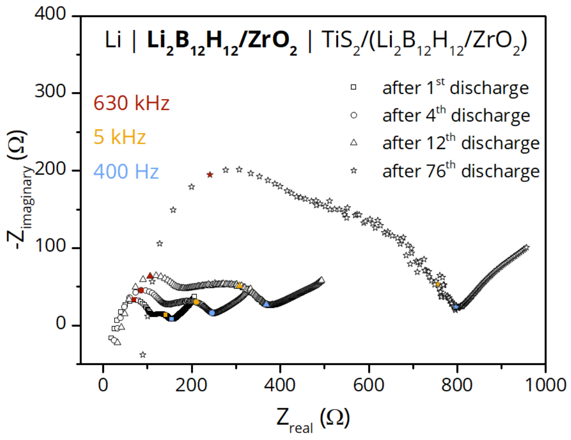

Figure S10. EIS data measured at 60 °C after certain charge/discharge cycles of the Li | $Li_2B_{12}H_{12}$ + 25 vol% ZrO_2 | $TiS_2/(Li_2B_{12}H_{12}$ + 25 vol% $ZrO_2)$ cell.

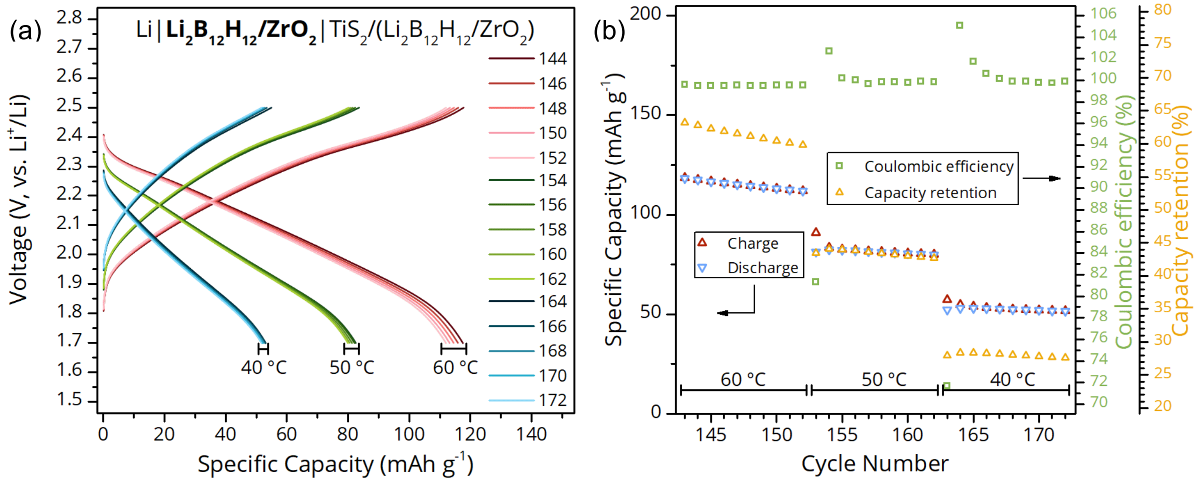

Figure S11. Battery performance of a $Li|Li_2B_{12}H_{12}$ + 25 vol% $ZrO_2|TiS_2$ cell, cycled at 40 °C, 50 °C and 60 °C with a current corresponding to 0.1 C. (a) Selected voltage profiles and (b) charge/discharge specific capacity, coulombic efficiency and capacity retention as function of cycle number.

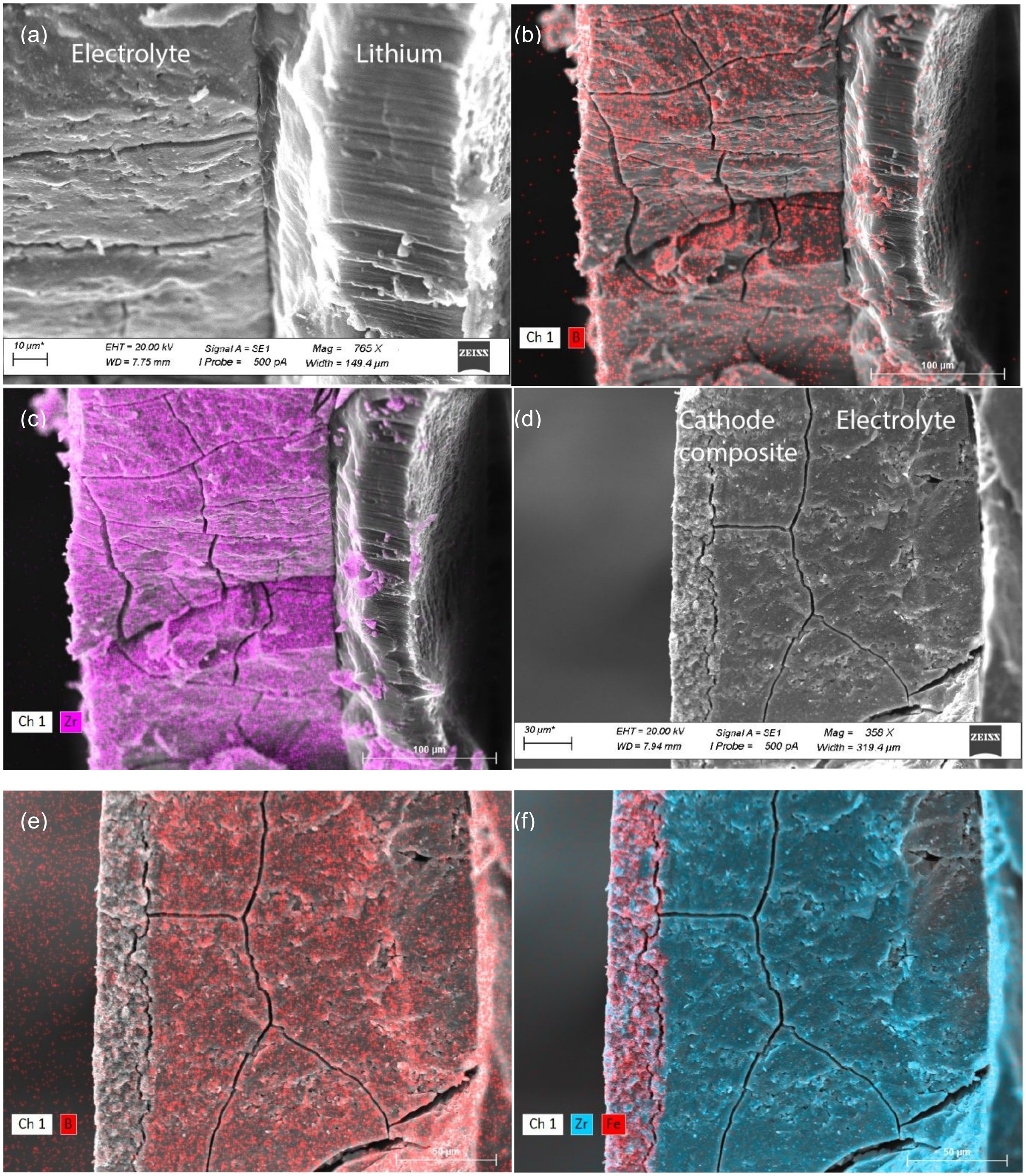

Figure S12. SEM cross sectional images of the $Li|Li_2B_{12}H_{12}$ + 25 vol% $ZrO_2|LiFePO_4/(Li_2B_{12}H_{12}$ + 25 vol% $ZrO_2)$ cycled battery pellet (a) Lithium metal/SSE interface and (b, c) corresponding elemental distribution maps. (d) Cathode composite/ SSE interface and (e, f) corresponding elemental distribution maps.

Scanning electron microcopy coupled with energy dispersive X-ray spectroscopy of the cycled battery pellet $Li|Li_2B_{12}H_{12}$ + 25 vol% $ZrO_2|LiFePO_4/(Li_2B_{12}H_{12}$ + 25 vol% $ZrO_2)$ as previously described[1], was adapted for use in this research with minor modifications. The cracks in the SSE visible in the SEM images are due to mechanical stress that occurred when the pellet was removed from the cell housing.

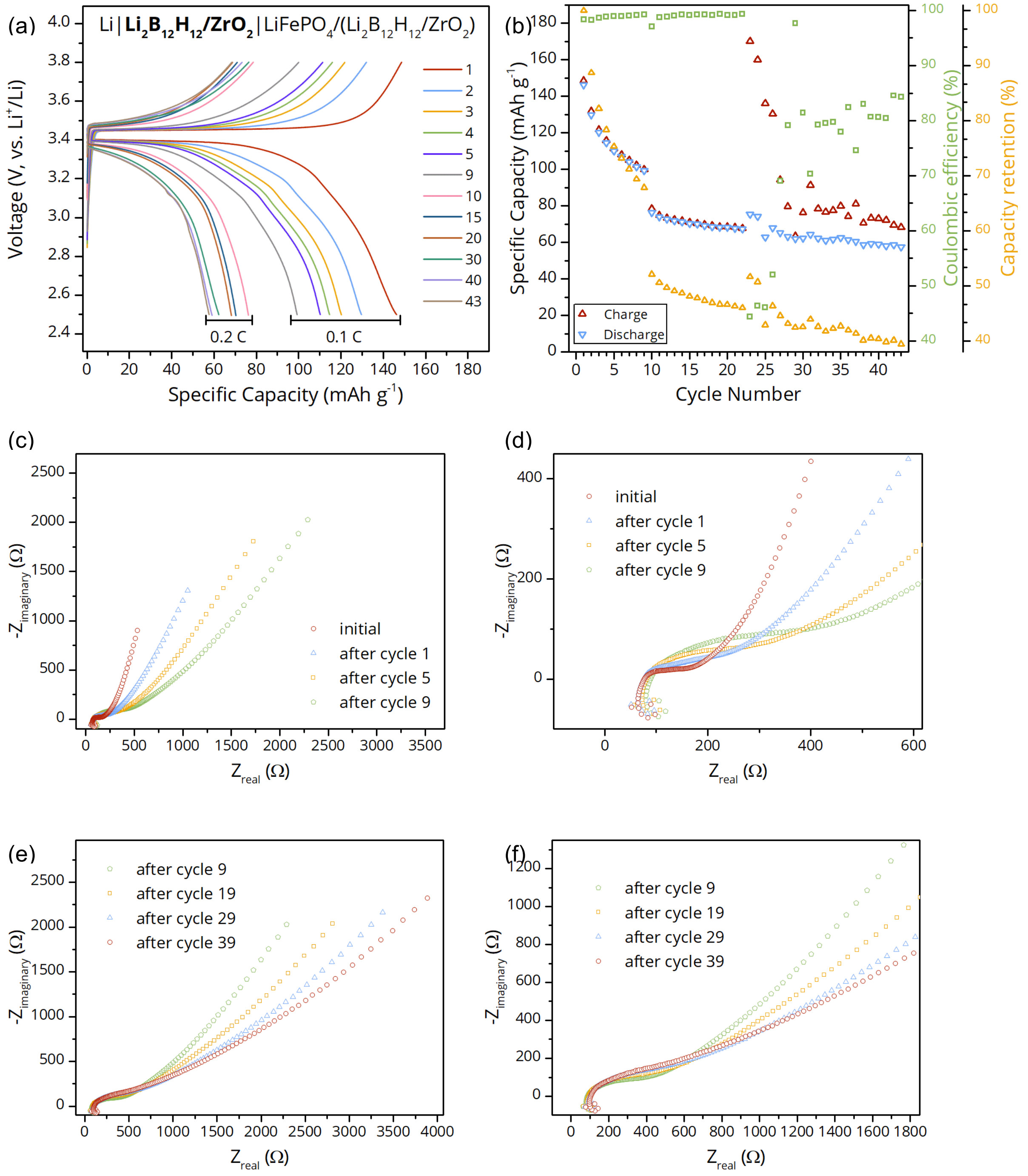

Figure S13. Battery data of a $Li|Li_2B_{12}H_{12}$ + 25 vol% $ZrO_2|LiFePO_4/(Li_2B_{12}H_{12}$ + 25 vol% $ZrO_2)$ cell, operated at C/10 and C/5 at a temperature of 60 °C. (a) Selected voltage profiles, (b) charge/discharge specific capacity, coulombic efficiency and capacity retention as a function of cycle number, (c,e) overview and (d,f) zoomed-in Nyquist plots.

Galvanostatic cycling of the $Li|Li_2B_{12}H_{12}$ + 25 vol% $ZrO_2|LiFePO_4/(Li_2B_{12}H_{12}$ + 25 vol% $ZrO_2)$ cell resulted in an exponential decrease in capacity and an increase in ohmic resistance during the initial 9 cycles at C/10. A high coulombic efficiency of averaged 98.9% was observed until cycle 22. Following an unknown event

in cycle 23, the coulombic efficiency dropped and stabilized at around 80% in the subsequent cycles. The cell impedance was monitored and did not significantly change after cycle 23.

References

(1) Chi, X.; Liu, H.; Xia, J.; Chen, H.; Yu, X.; Weng, W.; Zhong, S. Breaking the Fe3O4-wrapped copper microstructure to enhance copper–slag separation. *Int. J. Miner. Metall. Mater.* **2024**, *31* (10), 2312-2325. DOI: 10.1007/s12613-024-2861-4.
